# Yomix: an interactive tool for the exploration of low-dimensional embeddings in omics data

**DOI:** 10.1093/bioinformatics/btag301

**Published:** 2026-05-14

**Authors:** Nicolas Perrin-Gilbert, Nisma Amjad, Pierre Fumeron, Silvia Tulli, Joshua J Waterfall

**Affiliations:** Sorbonne Université, CNRS, Institut des Systèmes Intelligents et de Robotique (ISIR), Paris, F-75005, France; Sorbonne Université, CNRS, Institut des Systèmes Intelligents et de Robotique (ISIR), Paris, F-75005, France; Inserm U1330, Institut Curie Centre de Recherche, PSL University, Paris, France; Translational Research Department, Institut Curie Centre de Recherche, PSL University, Paris, France; Sorbonne Université, CNRS, Institut des Systèmes Intelligents et de Robotique (ISIR), Paris, F-75005, France; Inserm U1330, Institut Curie Centre de Recherche, PSL University, Paris, France; Translational Research Department, Institut Curie Centre de Recherche, PSL University, Paris, France

## Abstract

**Summary:**

In the analysis of diverse omics data, a common and important preliminary step involves computing low-dimensional embeddings using techniques such as PCA, UMAP, t-SNE, or variational autoencoders. These embeddings provide a global overview of sample distributions and their relationships, often serving as the basis for formulating biological hypotheses. To facilitate rapid and intuitive exploration of such low-dimensional embeddings, we developed Yomix, an interactive omics-agnostic visualization and data exploration tool. Yomix enables users to flexibly define subsets of interest using a lasso selection tool, instantly compute their feature signatures, and compare their distributions. Yomix is a fast and efficient tool for interactive exploration of diverse omics datasets.

**Availability and implementation:**

Yomix and its documentation are publicly available at https://github.com/perrin-isir/yomix.

## 1 Introduction

The high-dimensional nature of omics datasets presents substantial challenges in data interpretation. To facilitate visualization and exploration of such datasets, a widely adopted preliminary step is the computation of low-dimensional embeddings, typically in two or three dimensions (Becht et al. 2019, Moon et al. 2019). Dimensionality reduction techniques, such as Principal Component analysis (PCA), Uniform Manifold Approximation and Projection (UMAP) (McInnes et al. 2018), t-distributed Stochastic Neighbour Embedding (t-SNE) (van der Maaten 2009), and variational autoencoders (VAEs) (Lopez et al. 2018), are frequently employed for this purpose, offering advantages in capturing both global and local data features. These low-dimensional representations serve as a basis for deriving biological insights, enabling researchers to identify clusters (Kiselev et al. 2019), infer developmental trajectories (Saelens et al. 2019), and formulate hypotheses regarding underlying biological processes (Trapnell et al. 2014).

Despite the widespread adoption of dimensionality reduction techniques in omics workflows, effectively interpreting the resulting low-dimensional embeddings remains a significant challenge. In practice, researchers must frequently explore these embeddings iteratively, zooming into local regions, selecting subpopulations of interest, and querying their molecular signatures. This type of visual exploration is essential for identifying novel cell types, validating marker features (genes, CpG sites, k-mers, etc.). However, most existing analysis frameworks (e.g. Seurat (Hao et al. 2021), Scanpy (Wolf et al. 2018)) rely on static visualizations that are not directly responsive to user interaction or dynamic filtering. This restricts the user’s ability to adjust views, subset groups, or overlay metadata in real time, often requiring time-consuming re-computation or manual scripting for even basic exploratory tasks. While recent tools have been developed to address some of these issues (Li et al. 2020, Kotliar and Colubri 2021, Keller et al. 2025) there remains a significant need for fast, flexible approaches to interact with diverse types of omics datasets.

Yomix addresses these challenges through several capabilities not combined in existing tools: a lasso selection tool enabling free-form cluster definition directly on any embedding, with advanced options such as intersection, union and difference, native support for 3D embeddings and user-controlled axis selection across higher-dimensional spaces, interactive annotation capability, and sub-second signature computation across diverse omics data types. A detailed comparison of Yomix with existing interactive visualization frameworks: including scX (Waichman et al. 2024), iSEE (Rue-Albrecht et al. 2018), CELLxGENE (Abdulla et al. 2025), ShinyCell (Ouyang et al. 2021), ASAP (David et al. 2020), SEQUIN (Weber et al. 2023), and SCHNAPPS (Jagla et al. 2021), is provided in Supplementary Fig. 1, available as supplementary data at *Bioinformatics* online, highlighting the specific exploratory workflows enabled by Yomix.

To address these challenges, we developed Yomix, a lightweight yet powerful visualization framework that supports interactive projection views, metadata integration, and feature ranking across a variety of omics data types.

## Materials and methods

Yomix is a lightweight tool used to identify biologically relevant structures and discriminative features across multiple datasets. It is developed in Python and uses the Bokeh framework, which generates a JavaScript-based graphical interface and callbacks that run in the web browser. It requires an anndata object (Virshup et al. 2024) saved in .h5ad format as input which contains a data matrix and at least one low-dimensional embedding.

**Figure 1:**
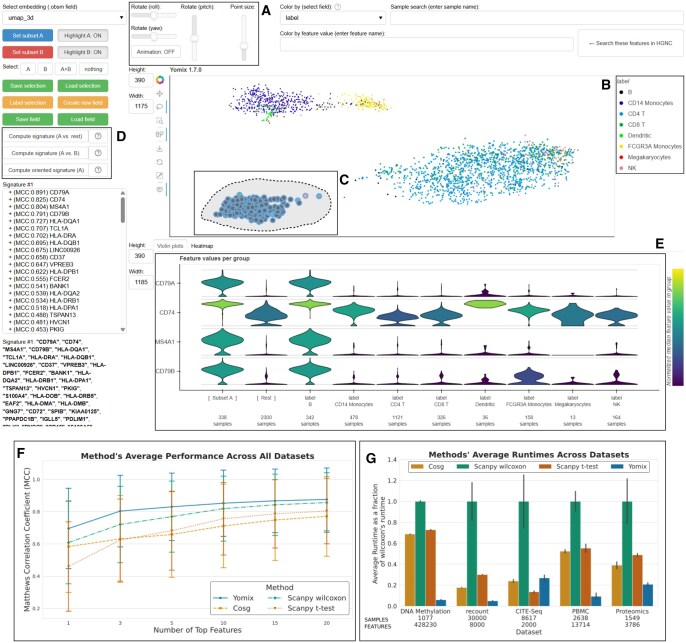
Screenshot of Yomix’s interface displaying the PBMC dataset after computing the signature of the bottom-left cluster, mostly composed of B cells. (A) Buttons to control real-time 3D rotation, and to interact in 3D with the scatter plot. (B) Users can click on legend to select data. (C) Lasso tool for manual selection of data points. (D) Users can compute different types of signatures. (E) Violin plots to visualize the distribution of selected features for selected subgroups. Users can switch to a heat map format. (F) Average performance comparison between SVM trained on features selected by different methods, error bars represent standard deviation. G) Runtime comparison between the different methods, error bars represent standard deviation.

By launching Yomix from the Python session, it directly opens an interactive plot of the embedding chosen by the user (Fig. 1A–E). Users can switch between different embeddings, change plot sizes and labels, and define specific subsets (Fig. 1A). These subsets can be selected using the legend and predefined annotations (Fig. 1B) or manually with a lasso tool (Fig. 1C), making it highly interactive. Yomix is both omics agnostic and handles three-dimensional (3D) embeddings. It also supports embeddings with more than 3 dimensions by letting users pick amongst dimensions for every axis.

Importantly, Yomix is not restricted to t-SNE or UMAP: it supports any low-dimensional embedding method, such as PCA, VAE (Lopez et al. 2018) or diffusion maps (Moon et al. 2019). Users should be aware that low-dimensional embeddings inevitably distort geometries, and structures visible in reduced dimensions, although often relevant, can sometimes be misleading. But there are tools that specifically try to avoid as much as possible those distortions, such as Γ-VAEs (Kim et al. 2024) which regularize geometric curvature. Lasso selections reflect the displayed layout rather than the full high-dimensional structure. The higher the embedding dimensionality, the lower the geometry distortion, so when possible we recommend relying on 3D embeddings. Yomix is fully functional with 3D and its original 3D lasso selection enables users to inspect data just as they would with 2D embeddings. Yomix also supports higher-dimensional embeddings, allowing users to interactively select 3 axes at a time to observe the data from many different perspectives.

Yomix can identify signatures for a user-defined cluster against the remaining data or between two user-defined subsets (Fig. 1D). These signatures, or marker features, are computed using a rapid, two-step filtering process that identifies the most discriminative features.

The algorithm first computes group statistics for each feature, calculating mean and standard deviation for Group A and Group B (either a specific subset or the remaining samples). It then calculates the Wasserstein distance between distributions for each feature, ranks all features in descending order by Wasserstein distance, and selects the features with the highest distributional differences. Wasserstein distances are computed using the closed-form solution for Gaussian distributions parameterized by the empirical mean and standard deviation of each group, and all operations are vectorized using NumPy, enabling sub-second runtimes even on large datasets. The second step identifies the features among the pre-filtered set that most reliably distinguish between groups using optimal binary classification thresholds. Specifically, for each feature, the algorithm tests multiple thresholds across the range of feature values, computes the confusion matrix for each threshold, calculates the Matthews Correlation Coefficient (MCC) at each threshold, and selects the one that maximizes MCC. The computation is vectorized across all features and thresholds simultaneously for efficient computation. A key advantage of this approach is its speed and accuracy, as filtering reduces the search space, and vectorized operations accelerate the search of optimal thresholds. More details on this implementation are available in Yomix documentation https://perrin-isir.github.io/yomix/

In addition, Yomix can compute oriented signatures by letting the user select a subset and draw an arrow in the embedding space. Each element of the subset is projected onto the arrow, creating a vector of positions along the arrow. For every feature, the algorithm computes the Pearson correlation between the feature values and the projection vector. It then returns the top features sorted by highest correlation. Selected features, either from signatures or input by the user, are visualized separately as either violin plots or heatmaps (Fig. 1E). Signatures display the top 100 features, providing a sufficiently large feature set for downstream analyses such as pathway enrichment or transcription factor activity inference. Analyses were run on a Dell desktop computer equipped with 32.0 GiB of RAM and a 13th Gen Intel® Core™ i9-13950HX processor.

## Results

To evaluate the predictive and computational performance of Yomix, we compared it with three widely used feature selection methods: *t* test, Wilcoxon test, and COSG, which was shown to perform well in a recent benchmarking study (Pullin and McCarthy 2024). COSG is a feature selection method that evaluates features by their geometry in sample space (Dai et al. 2022). For each group, it constructs an ideal feature, one that is expressed exclusively in the cluster of interest and not in any other sample. It then computes the cosine similarity between every feature vector and this ideal vector, returning features with the smallest angular distance to the ideal gene vector of the target group and the largest angular distance to the ideal vectors of other groups.

Benchmarks were performed on five diverse omics datasets that include bulk RNAseq from The Cancer Genome Atlas (TCGA) pan-cancer analysis (Hoadley et al. 2018) (10 541 samples, 8000 features), DNA methylation array profiling of over 1000 sarcomas (Koelsche et al. 2021), single cell multiomics of cord blood (Stoeckius et al. 2017), scRNAseq of peripheral blood mononuclear cells (PBMCs) (Hansen et al. 2023) and proteomics from the National Cancer Institute’s Clinical Proteomic Tumor Analysis Consortium (CPTAC) (Li et al. 2023). Dataset sizes and feature counts are detailed in Fig. 1.

### Preprocessing

We used the raw proteomics data. Recount and CITE-seq were log-normalized and subset to the 8000 and 2000 highly variable genes respectively. PBMC was log-normalized as well, but all the available genes were kept for analysis. Regarding the DNA methylation dataset, we followed the procedure described in the original publication (Koelsche et al. 2021).

To evaluate the utility of features selected by the various methods, we computed signatures for one cluster against the remaining samples (A vs Rest) using the 4 different methods (Yomix, *t* test, Wilcoxon test, and COSG). This task was repeated for every cluster for every dataset. In Recount, the clusters are the projects if the samples came from TCGA or the tissues of origin if they came from GTEX. For PBMC and CITE-seq, the goal is to predict the cell type and activation state. In DNA methylation and proteomics datasets, the aim is to predict the cancer type. We selected an increasing number of top features (from 1 to 20) from each method and measured the average MCC using support vector machines (SVM) as the classifier with default parameters on these features across all datasets. Each classifier is trained on 70% of the dataset, the remaining 30% are used as a test set. The train and test are randomly sampled and the frequencies of the two classes (A and Rest) are preserved. We assessed feature signature quality using MCC scores on the test set from the SVM classification. Benchmarking code is available at https://github.com/PierreFumeron/yomix_benchmark.

The results shown in Fig. 1 demonstrate that Yomix consistently selects feature signatures with the highest predictive power (Fig. 1F). Yomix achieved average MCC scores comparable to or exceeding those of other methods across nearly all feature set sizes, underscoring its ability to select discriminative and biologically informative markers. Wilcoxon was the second best performing method, while COSG and *t* test yielded feature sets with lower MCC scores. All methods showed improved performance when more features were included. Similar results were obtained using KNN and Random Forest classifiers as shown in Supplementary Fig. 2, available as supplementary data at *Bioinformatics* online, confirming that these findings are not specific to the choice of classification model.

As a concrete biological illustration, Fig. 1 displays the PBMC scRNAseq dataset with Yomix’s signature computed for the B cell cluster (lasso-selected in panel C) against all remaining cells. The top-ranked features include well-established B cell markers such as CD79A, CD74, MS4A1, and CD79B, whose preferential expression in B cells relative to all other cell populations is clearly visible in the violin plots (Fig. 1E).

In addition to predictive performance, speed was also computed for each method. The shortest runtimes in all datasets were also attained by Yomix, with execution times remaining below 0.7 seconds even for the largest datasets (Fig. 1G). When analyzing the TCGA bulk RNAseq dataset, which has 10 541 samples and 8000 features, the signature computation was completed in approximately 0.165 seconds. In contrast, the Wilcoxon method exhibited the longest runtime, 5.89 seconds on methylation and 2.99 seconds on TCGA datasets. COSG and *t* test methods displayed intermediate performance as they outperformed Wilcoxon but remained slower than Yomix across all datasets. The results demonstrate the significant computational efficiency of Yomix. These findings highlight the scalability and speed of Yomix’s two-step algorithm, making it particularly well-suited for the rapid, iterative analysis required in large-scale omics exploration.

This analysis confirms that Yomix not only offers superior computational speed and interactivity but also excels at identifying feature sets with notable biological relevance and predictive capability, regardless of the type of dataset used and the size of samples required.

To further benchmark Yomix in the context of data types for which specialized methods exist, we performed two additional comparisons. For bulk RNA-seq, we compared Yomix against pyDESeq2 on a randomly subsampled subset of 50% of the TCGA dataset (5272 samples, 8000 highly variable genes), performing differential analysis for each of the 33 TCGA project types against the remaining samples. Results are shown in Supplementary Fig. 3, available as supplementary data at *Bioinformatics* online; while pyDESeq2 required an average of 6 minutes per project comparison, Yomix completed the equivalent task in under one second. For proteomics, we compared Yomix against ProMS  (Shi et al. 2021), a method specifically designed for protein biomarker discovery. As shown in Supplementary Fig. 4, available as supplementary data at *Bioinformatics* online, the two methods yield comparable feature selection performance on the CPTAC proteomics dataset.

Taken together, these results demonstrate that Yomix offers strong computational efficiency, seamless interactivity, and feature selection performance that is competitive with established methods across diverse omics data types. While the benchmarking presented here covers five datasets spanning five data types, we consider these representative use cases rather than exhaustive proof of universal superiority; the primary strengths of Yomix lie in its speed and the interactive workflows it uniquely enables.

## Supplementary Material

btag301_Supplementary_Data

## Data Availability

Yomix and its documentation are publicly available at https://github.com/perrin-isir/yomix. Benchmarking code is available at https://github.com/PierreFumeron/yomix_benchmark. scRNAseq of peripheral blood mononuclear cells (PBMCs) is available at https://bioconductor.org/packages/TENxPBMCData/.
