# Supplementary material for: Yomix: an interactive tool for the exploration of low-dimensional embeddings in omics data
Source: Bioinformatics. 2026 May 14;42(5):btag301. doi: 10.1093/bioinformatics/btag301 (PMC13198385; doi:10.1093/bioinformatics/btag301)

Supplementary Figures

Yomix: An Interactive Tool for the Exploration of Low-Dimensional Embeddings in Omics Data

Supplementary Figure 1: Comparative table

	YOMIX	Sciviewer	Vitessce	scX	ISEE	CellxGene	ShinyCell	ASAP	SEQUIN	SCHNAPPS
Currently maintained (update in the past year)	☑	☐	☑	☐	☑	☑	☑	☑	☐	☑
Marker signatures for a user-defined subset (A vs rest)	☑	☑	☐	☑	☐	☑	☐	☑	☑	☑
Marker signatures for 2 user-defined subsets (A vs B)	☑	☐	☐	☐	☐	☑	☐	☑	☑	☑
Oriented signatures	☑	☑	☐	☐	☐	☐	☐	☐	☐	☐
Filter data by marker (feature) expression	☑	☐	☑	☐	☑	☑	☐	☐	☑	☑
Advanced lasso selection (union, intersection, 3D ...)	☑	☐	☐	☐	☐	☐	☐	☐	☐	☐
Interactive legend	☑	☐	☑	☑	☐	☑	☐	☑	☐	☑
heatmap / violin plots / histogram : distribution of metadata variable	☑	☑	☑	☑	☑	☑	☑	☑	☑	☑
3D interactive scatter plots	☑	☐	☑	☑	☐	☐	☐	☑	☐	☑
Annotate	☑	☐	☑	☐	☐	☑	☐	☑	☑	☑
Bivariate plot (co-expression)	☐	☐	☑	☑	☑	☑	☑	☑	☐	☑
Hover option	☑	☑	☑	☑	☐	☐	☐	☑	☑	☐
Sample search	☑	☐	☐	☐	☑	☐	☐	☐	☐	☑
Feature search	☑	☑	☑	☐	☑	☑	☑	☑	☑	☑
Omics agnostic	☑	☐	☑	☐	☐	☐	☑	☐	☐	☐
accelerated signature computation	☑	☐		☐		☐		☐	☐	☐
Color by sum/mean/median of features (feature arithmetics)	☑	☐		☐		☑		☐	☑	☐

Supplementary Figure 2: KNN/RF classifiers

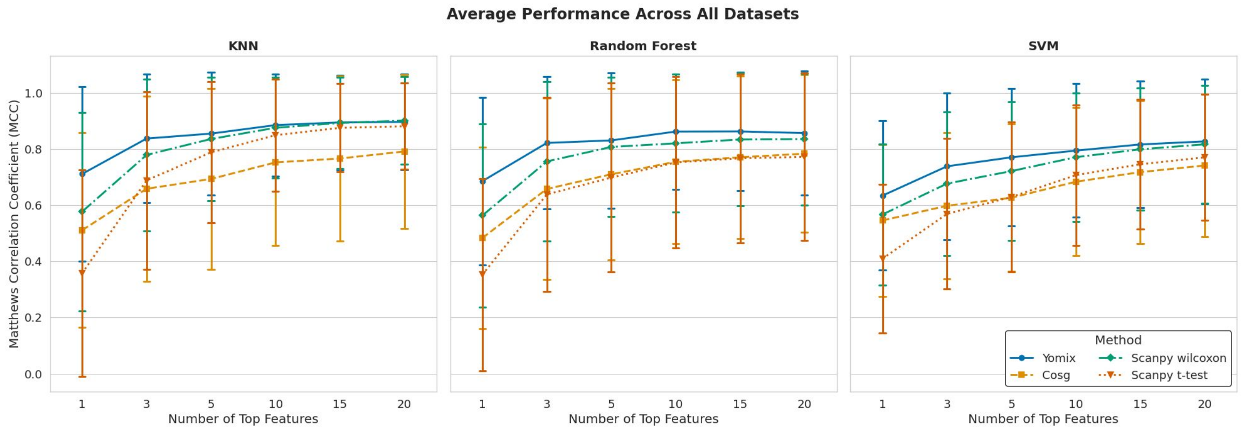

Supplementary Figure 3: Volcano plots of DEGs in TCGA cohort

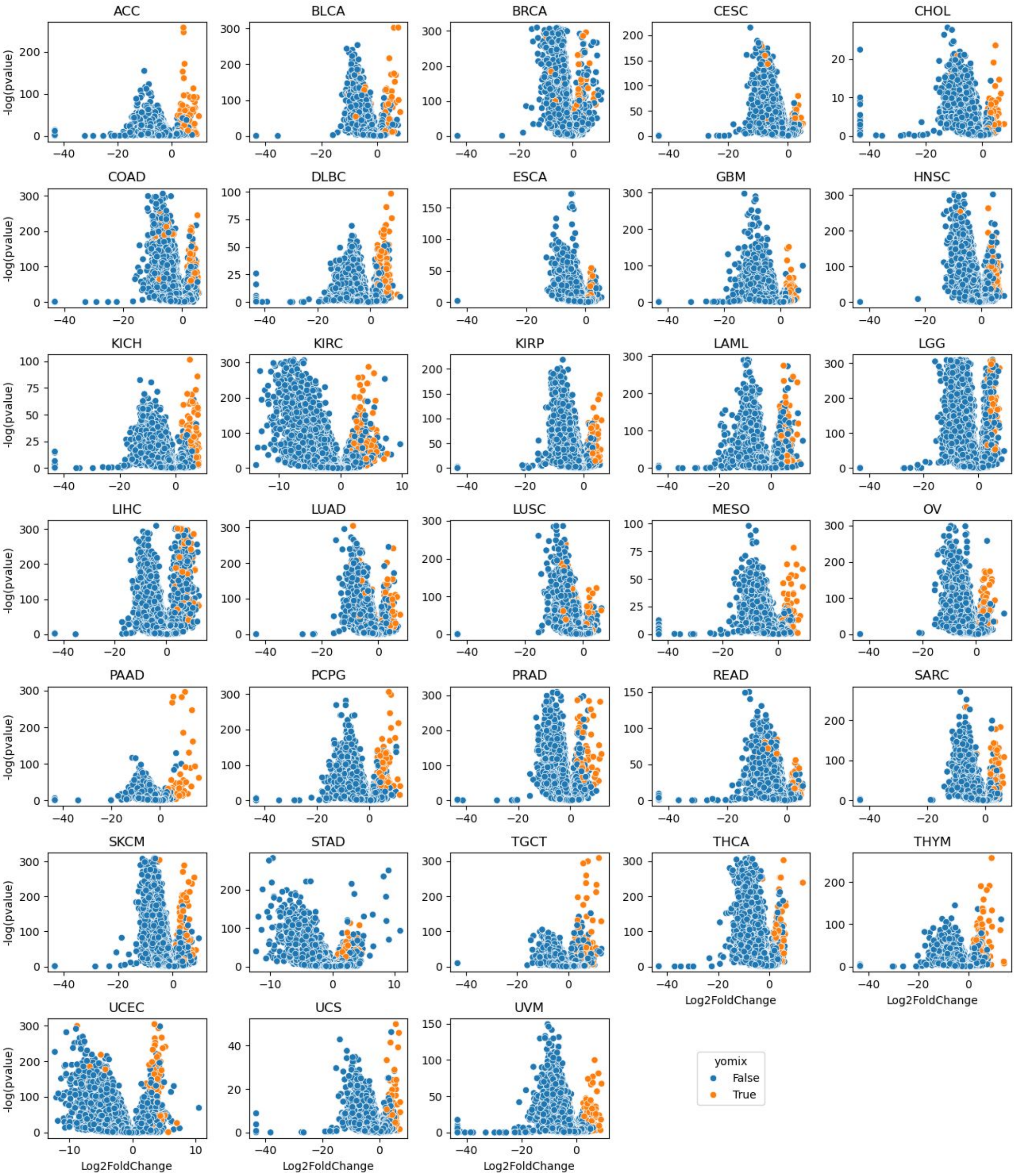

Supplementary Figure 4: PROMS comparison

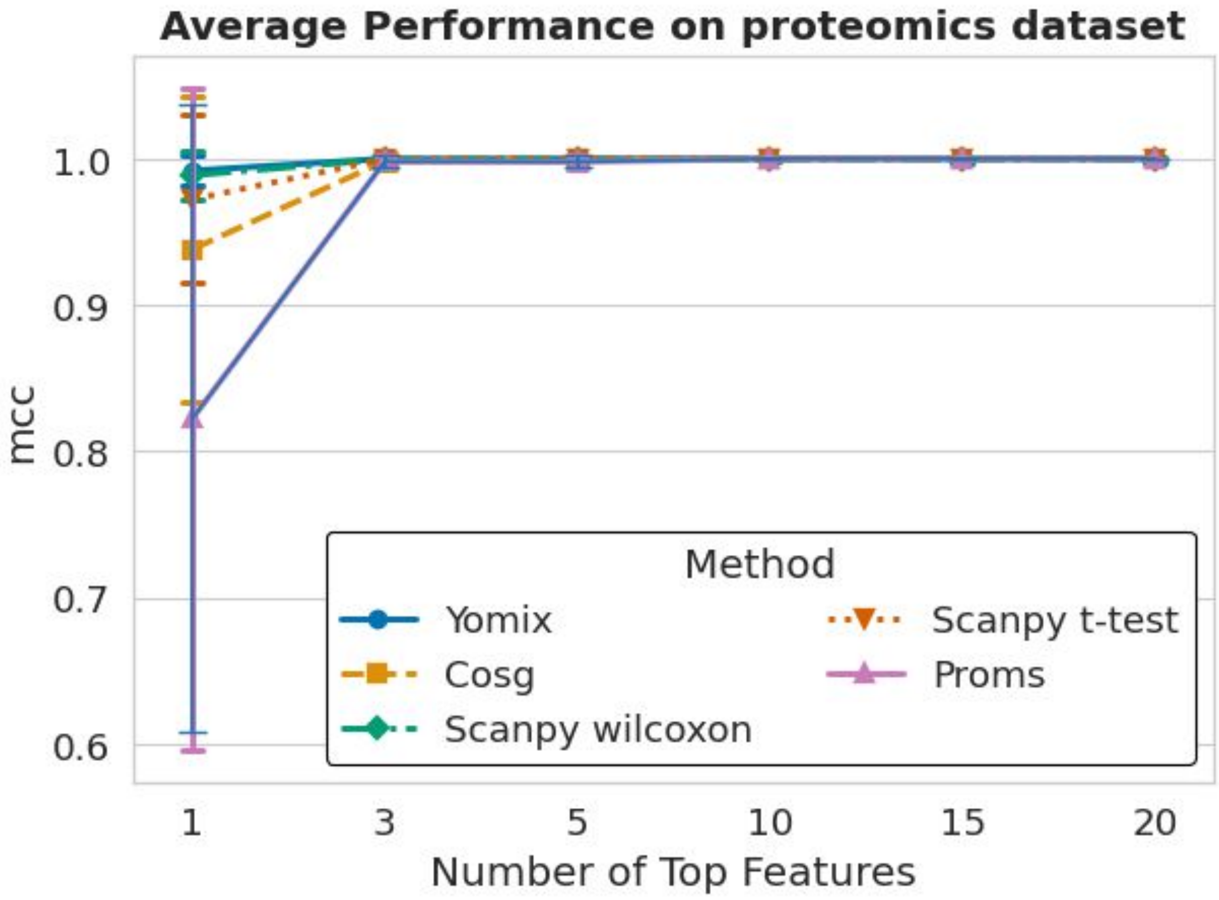

Supplement: btag301_Supplementary_Data [file btag301_supplementary_data.pdf]
